# Functional Characterization of a Hydroxyacid/Alcohol Hydroxycinnamoyl Transferase Produced by the Liverwort *Marchantia emarginata*

**DOI:** 10.3390/molecules22111854

**Published:** 2017-10-31

**Authors:** Ping-Ping Wang, Hui Liu, Shuai Gao, Ai-Xia Cheng

**Affiliations:** Key Laboratory of Chemical Biology of Natural Products, Ministry of Education, School of Pharmaceutical Sciences, Shandong University, Jinan 250012, China; wangpingp1234@163.com (P.-P.W.); ttklliuhui@163.com (H.L.); gaoshuai.3331166@163.com (S.G.)

**Keywords:** ferulate esters, BAHD, acyltransferase, cutin, bryophytes

## Abstract

The aerial organs of most terrestrial plants are covered by a hydrophobic protective cuticle. The main constituent of the cuticle is the lipid polyester cutin, which is composed of aliphatic and aromatic domains. The aliphatic component is a polyester between fatty acid/alcohol and hydroxycinnamoyl acid. The BAHD/HxxxD family enzymes are central to the synthesis of these polyesters. The nature of this class of enzymes in bryophytes has not been explored to date. Here, a gene encoding a fatty ω-hydroxyacid/fatty alcohol hydroxycinnamoyl transferase (HFT) has been isolated from the liverwort *Marchantia emarginata* and has been functionally characterized. Experiments based on recombinant protein showed that the enzyme uses ω-hydroxy fatty acids or primary alcohols as its acyl acceptor and various hydroxycinnamoyl-CoAs—preferentially feruloyl-CoA and caffeoyl-CoA—as acyl donors at least in vitro. The transient expression of a *MeHFT-GFP* fusion transgene in the *Nicotiana benthamiana* leaf demonstrated that MeHFT is directed to the cytoplasm, suggesting that the feruloylation of cutin monomers takes place there.

## 1. Introduction

The evolution of plants from marine/aquatic to terrestrial organisms has necessitated the development of a protective outer coat, primarily to prevent water loss [1,2,3]. The material making up this coat typically contains substantial quantities of the polymers cutin and suberin [1,4,5]. Cutin has a high content of the C16 fatty acid ferulic acid and the C18 fatty acid *p*-coumaric acid linked by esterification to glycerol [1,5,6]. Although cutin is primarily an acylglycerol polymer, varying amounts of certain aromatics (ferulic acid, coumaric acid and caffeic acid) feature in some cutins [1].

The enzymes responsible for acylation belong to the large BAHD/HXXXD acyltransferase family [7], almost all the members of which harbor both the HxxxD and the DFGWG motif. The HxxxD sequence is thought to be involved in enzyme catalysis [8], the DFGWG motif is distal to the active site in space and appears to have a structural function [9,10]. Three functional classes of these enzymes have been identified, namely the alcohol acetyltransferases [11,12,13], the anthocyanin/flavonoid acyltransferases [14,15,16,17,18] and the hydroxycinnamoyl transferases [19,20,21]. Their molecular masses range from 48 to 55 kDa [10]. The enzyme hydroxycinnamoyl-CoA:ω-hydroxyacid/fatty alcohol transferase (HHT) catalyzes the formation of aromatic esters in lipidic polymers. Its activity has been noted in many plant species, both angiosperms and gymnosperms [22,23]. The *Arabidopsis thaliana* BAHD/HXXXD family acyltransferase, encoded by the gene *At5g41040*, has been shown to have HHT activity [20,21] and is employed in the formation of suberin aromatics. The suppression of a potato homolog of *At5g41040* reduces both the ferulate ester and ω-hydroxy fatty acid contents of suberin, resulting in the development of a thickened peel and an increased rate of water loss [24]. In contrast, an *A. thaliana* mutant lacking cutin feruloyltransferase (a distant homolog of HHT1) activity displays no marked alteration in its cuticle function in relation to ion permeability, water loss or pathogen infection [25]. 

The *Populus trachocarpa* FHT1 enzyme accepts both *p*-coumaroyl and feruloyl CoA as a thioester donor and is able to acylate ω-hydroxy acids and fatty alcohols in vitro. Heterologously expressing the *Populus* gene in *A. thaliana* has the effect of increasing, by as much as 45%, the incorporation of phenolic esters into both the leaf cutin and the root and seed suberin; the accumulation of ferulate esters conferred thereby confer a significantly increased tolerance to salinity stress [26]. WoLF PSORT analysis [27] has indicated that FHT is most likely a cytosolic enzyme, as no signal peptides for targeting the protein to organelles or transmembrane domains have been recognized [24].

Bryophytes, the group of non-vascular plants which includes mosses, liverworts and hornworts, were among the first plants to colonize land. To achieve this, they needed to evolve an number of adaptations to cope with a range of abiotic stresses which are not relevant in a marine or aquatic environment [28]. The liverworts synthesize a wide range of terpenoids [29], lignin [30], flavonoids [31] and bis-bibenzyls [32,33]. As yet, however, their synthesis of cutin has not been described. Here, a HFT enzyme produced by the liverwort species *M. emarginata* (MeHFT) has been characterized. The enzyme appears to be involved in the formation of alkyl hydroxycinnamate esters and is active in the cytoplasm; in vitro, the enzyme can use either feruloyl or caffeoyl-CoA as its acyl donor, and ω-hydroxyacids or fatty alcohols as its acyl acceptor. 

## 2. Results and Discussion

### 2.1. Gene Isolation and Sequence Analysis 

**Figure 1:**
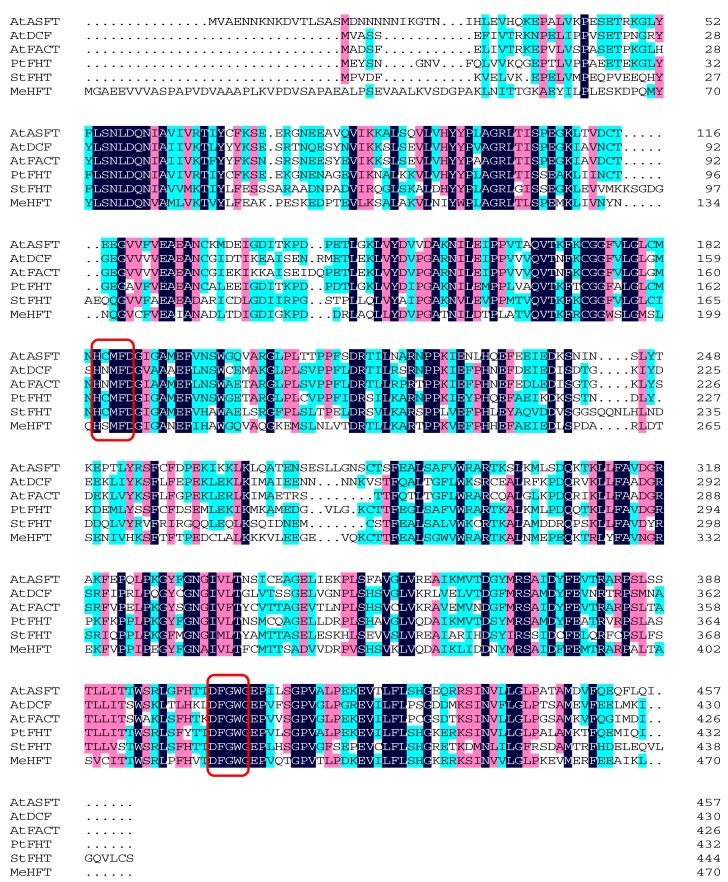
Sequence alignment of MeHFT with PtFHT from *P. trichocarpa* (JX515962), StFHT from *S. tuberosum* (ACS70946), AtASFT (AT5G41040), AtDCF (AT3G48720) and AtFACT (AT5G63560) from *A. thaliana*. The conserved HxxxD and DFGWG motifs are indicated by red boxing.

**Figure 2:**
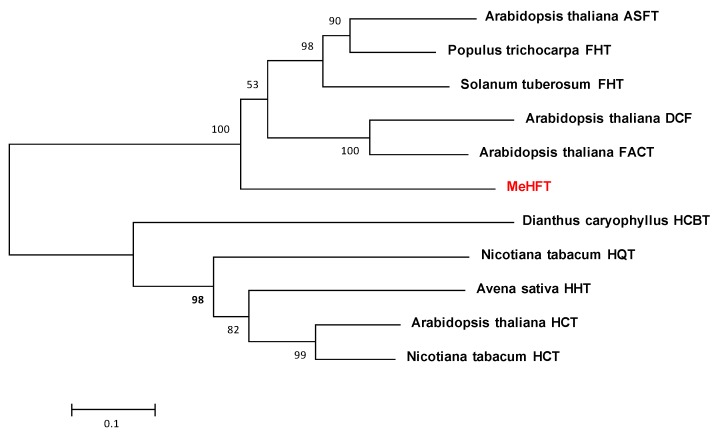
A phylogenetic analysis of MeHFT. The Uniprot accession numbers of the proteins included in the analysis are: *Populus trichocarpa* FHT (JX515962), *Solanum tuberosum* FHT (ACS70946), *A. thaliana* ASFT (AT5G41040), *Dianthus caryophyllus* HCBT (CAB06430), *A. thaliana* DCF (AT3G48720), *A. thaliana* FACT (AT5G63560), *N. tabacum* HQT (CAE46932), *Avena sativa* HHT (BAC78633), *A. thaliana* HCT (AT5G48930), *N. tabacum* HCT (CAD47830).

On the basis of the Swissprot in silico annotation of the *M. emarginata* transcriptome (SRP078649)*,* a sequence encoding a putative HFT was identified and given the designation *MeHFT*. The sequence’s length was 1413 nt and its predicted product a 470 residue protein of molecular mass 51.93 kDa and pI 4.91. The predicted polypeptide’s sequence shared, respectively, 50.6%, 48.2%, 42.3%, 44.3% and 49.5% identity with those of PtFHT (JX515962: [26]), AtHHT1/ASFT (AT5G41040: [20,21]), AtDCF (AT3G48720: [25]), AtFACT (AT5G63560: [34]) and StFHT (ACS70946: [24]). Sequence alignments confirmed that each these proteins harbored the HxxxD and DFGWG motifs (Figure 1). A phylogenetic analysis (Figure 2) of the *M. emarginata* sequence suggested the existence of two distinct clades: the first grouped PtFHT1, StFHT, AtHHT1/ASFT, AtFACT and AtDCF, while the second grouped DcHCBT, NtHQT, AsHHT, AtHCT and NtHCT. The MeHFT sequence was related to the former clade; its peripheral position suggested that its function may involve the formation of polyester aromatics. Finding this gene is such a basal land plant implies that it likely represents an ancestral sequence of homologs present in vascular plants.

### 2.2. Functional Analysis

**Figure 3:**
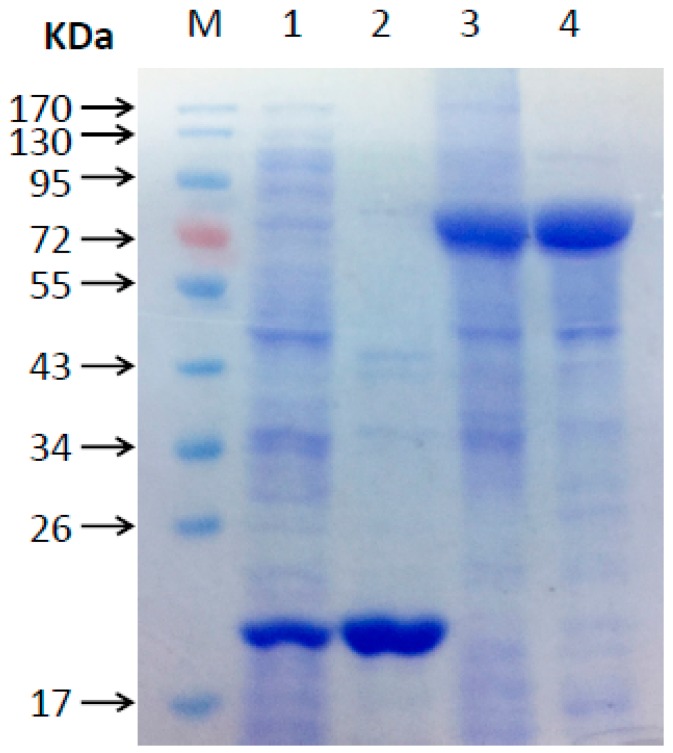
SDS-PAGE separation of recombinant MeHFT. Lane M: molecular mass standards. Lane 1: culture medium from *E coli* cells harboring an empty pET32a vector control; lane 2: proteins purified from the culture medium used in lane 1; lane 3: culture medium from *E coli* cells harboring pET32a-MeHFT; lane 4: proteins purified from the culture medium used in lane 3.

**Figure 4:**
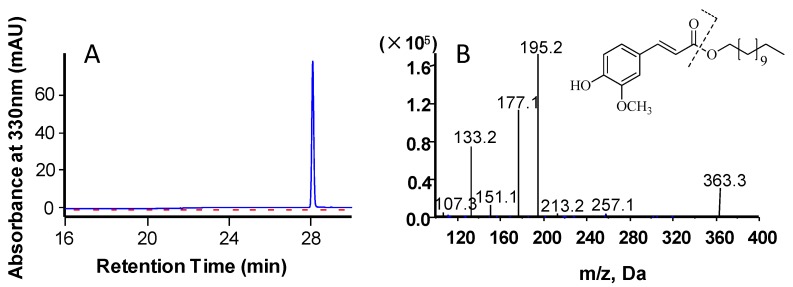
In vitro activity of MeHFT recombinant enzymes. (**A**) HPLC separation of the reaction products of recombinant MeHFT (the solid blue line) or the negative control (empty vector harboring cells) (the red dashed line) provided with feruloy-CoA and 1-dodecanol; (**B**) The MS/MS spectrum of the alky ferulate product from (**A**).

The molecular mass of the recombinant MeHFT protein, as estimated from its migration through an SDS polyacrylamide gel was ~71 kDa (including the 20.4 kDa His-tag) (Figure 3), which is in line with that of most BAHD acyltransferases. The experiments run to test its ability to accept a range of thioester donors (*p*-coumaroyl CoA, caffeoyl CoA and feruloyl CoA) and acyl acceptors (16-hydroxypalmitic acid, hexadecanoic acid and a range of primary alcohols [C6, C7, C10, C12, C14, C16, C20, C22]) showed that when recombinant MeHFT was provided with feruloyl CoA and 1-dodecanol, a single prominent HPLC peak was observed (Figure 4A). MS/MS analysis of this reaction product revealed it to be a molecular ion [M+H]^+^ with an *m/z* of 363.3, consistent with it being a complex of ferulate with C12 (Figure 4B). The presence of an additional fragment with an *m/z* of 177.1 implied the presence of a feruloyl moiety in the reaction product. No products were generated from a control reaction run with the protein obtained from the culture of *E. coli* carrying an empty pET32a plasmid (Figure 4A). When C10 was provided as the acyl acceptor, the activity of the recombinant MeHFT provided with caffeoyl CoA as a substrate was only ~38% of that recorded when feruloyl CoA was provided and ~43% when using *p*-coumaroyl CoA (Table 1). The data implied that the preferred acyl donor of MeHFT was feruloyl CoA. With feruloyl-CoA as the acyl donor, a comparison of 15 aliphatic substrates as potential acyl acceptors (Table 1) showed that 1-decanol, 1-dodecanol and 1-tetradecanol were similarly effective as acceptors. The recombinant enzyme showed a level of activity when provided with the short-to-mid-chain fatty alcohols (C6, C7, C10, C12, C14) but none when provided with the long chain fatty alcohols (C20, C22). 

Experiments designed to identify the optimal reaction conditions for recombinant MeHFT showed that the enzyme preferred a temperature of 40 °C and a pH of 7.5. At this temperature and pH optimum, recombinant MeHFT, when provided with ~0.1 mM feruloyl-CoA as the acyl donor, was associated with comparable K_cat_/K_m_ ratios in the presence of 1-decanol (3181 M^−1^s^−1^) and 1-dodecanol (3015 M^−1^s^−1^) (Table 1). When provided with 0.1 mM 1-decanol and feruloyl-CoA the K_cat_/K_m_ rose to 3928 M^−1^s^−1^, a value comparable to that achieved by HFTs isolated from higher plants [26]. These measurements suggested that, in the presence of a non-limiting supply of CoA thioester, MeHFT is effective in catalyzing transferuloylation on aliphatic monomers, at least in vitro. Just as is the case with AtFACT, MeHFT recognized feruloyl-CoA, caffeoyl-CoA and *p*-coumaroyl-CoA as an acyl donor, although its preference was for feruloyl-CoA, while that of AtFACT was for caffeoyl-CoA [31]. The substrate preferences of AtHHT1/ASFT, StHFT and PtHFT are the same as that of MeHFT. 

### 2.3. The Subcellular Localization of MeHFT

**Figure 5:**
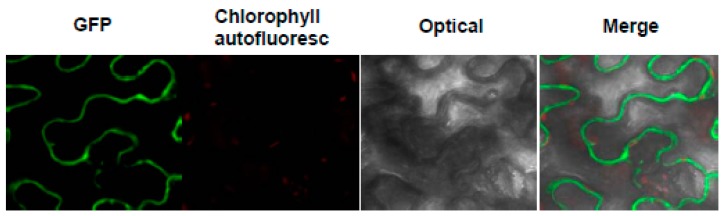
Expression of the p*35S::MeHFT-GFP* transgene in a transiently transformed *N. benthamiana* leaf reveals the localization of MeHFT to the cytoplasm. The GFP signal appears green and the chlorophyll auto-fluorescence signal red.

The pattern of expression of the p*35S::MeHFT-GFP* trangene in the *N. benthamiana* leaf is shown in Figure 5. The clear result was that the translation product was deposited in the cytoplasm. 

## 3. Materials and Methods

### 3.1. Plant Materials

*M. emarginata* thallus was raised under a 12 h photoperiod and a constant temperature of 25 °C. Harvested thalli were snap-frozen in liquid nitrogen and stored at −80 °C. *Nicotiana benthamiana* plants were soil-grown for 5–6 weeks under a 12 h photoperiod, with a day/night temperature regime of 24 °C/22 °C. 

### 3.2. Reagents

All chemicals and reagents required were purchased from either Sigma–Aldrich (St. Louis, MO, USA) or Energy Chemical (Shanghai, China), unless otherwise noted. Feruloyl CoA, *p*-coumaroyl CoA and caffeoyl CoA were synthesized from ferulic acid, *p*-coumaric acid and caffeic acid, respectively, using liverwort 4-coumarate CoA ligase [35], then purified using an LC-18 SPE column (Supelco Inc., Bellefonte, PA, USA), following the recommendation of Beuerle and Pichersky [36]. The products were checked for purity using a spectrophotometric assay.

### 3.3. Phylogenetic Analysis of the MeHFT Sequence

A phylogenetic analysis involving the *MeHFT* sequence and certain selected related plant proteins was carried out using routines implemented in MEGA v5.1 software. [37]. The Neighbor-Joining method was applied, and a bootstrap analysis, based on 1000 replicates, was performed to give statistical support for the derived clade structure. 

### 3.4. Gene Isolation, Recombinant Protein Expression and Purification

Total RNA from two month old *M. emarginata* thallus was extracted using a CTAB-based method [38], and reverse transcribed with the help of a PrimeScript™ RT Master Mix Kit (Takara Bio, Inc., Shiga, Japan) according to the supplier’s protocol. The full length cDNA produced by *MeHFT* was amplified from a cDNA template using PrimeSTAR HS DNA polymerase (Takara) driven by the primer pair *MeHFT-F/-R* (sequences given in Table 2). The full length clone was subcloned into pMD19-T (Takara, Japan) and then transformed into Escherichia coli DH5a. Plasmids from positive transformants were extracted with Plasmid Mini Kit (Omega Bio-Tek, Norcross, GA, USA) and fully sequenced. The plasmids was then double-digested with *SacI* and *XhoI* (Takara, Japan), and ligated into the corresponding cleavage sites of pET32a (Novagen, Darmstadt, Germany) in order to generate a transgene which encoded a His-tagged version of *MeHFT*. To heterologously express this transgene, it was transferred into competent *E. coli* BL21 (DE3) cells Expression of the transgene was induced by the addition of 0.5 mM isopropyl β-d-1-thiogalactopyranoside to the culture medium, and the cells were held at 16 °C for 16 h. Recombinant protein was purified from the medium by passage through a Ni-NTA Sefinose His-bind column (Bio Basic Inc., Markham, ON, Canada), following Gao et al. [35]. The concentrations and approximate molecular masses of the recombinant protein were determined as described elsewhere [39].

### 3.5. Enzymatic Assay and Kinetics Determination

Each 50 μL enzymatic assay reaction comprised 1 μg recombinant protein, along with 50 μM of either *p*-coumaroyl-CoA, caffeoyl-CoA or feruloyl-CoA and 20 μM acyl acceptor (see below) in 0.1 M Tris-HCl buffer, pH 7.5. The reactions were initiated by the addition of the recombinant protein, held at 40 °C for 20 min, and terminated by the addition of 50 μL acetonitrile. After centrifugation (12,000× *g*, 20 min) to remove any precipitated protein, a 20 μL aliquot of the mixture was injected into an HPLC device equipped with C18 reverse-phase column (4.6 × 250 mm Agilent, Santa Clara, CA, USA). The material was eluted by passing a mixture of 0.2% *v*/*v* glacial acetic acid in water (A) and acetonitrile (B) provided at a flow of 0.8 mL min^−1^. In the period 0–5 min, the eluate was composed of 85% A, 15% B; from 5 to 25 min, the proportion of B was raised linearly to 100%; from 25 to 35 min, the eluate was 100% B; and from 35 to 40 min, the proportion of B was lowered linearly to 15%. UV absorption was monitored at 330 nm.

The reaction kinetics of the recombinant MeHFT was tested in two sets of experiments. In the first, the recombinant enzyme was provided with 0.1 mM 1-decanol and a range of concentrations of feruloyl-CoA in 0.1 M Tris-HCl buffer (pH 7.5), while in the second, the feruloyl-CoA concentration was fixed at 0.1 mM and the 1-decanol concentration was varied. Both sets of experiment utilized 1 μg purified protein, and were held at 40 °C for 10 min. Kinetic data were calculated on the basis of the Michaelis-Menten equation, using Graphpad Prism 5 software (www.graphpad.com). The quantity of reaction product was calculated based on a standard calibration curve. 

### 3.6. The Subcellular Localization of MeHFT

The *MeHFT* sequence was inserted into pGWB5 (p*35S::GL2-GFP*) (www.thermofisher.com) for the purpose of determining the subcellular localization of MeHFT. After its amplification driven by primer pair attB1-MeHFT-F/R (Table 2), the sequence was introduced into pDONR207 (Invitrogen, Carlsbad, CA, USA) using the BP reaction, as recommended by the manufacturer. Correct insertion was verified by sequencing. Following validation by sequencing, the construct was transferred into pGWB5 via the LR reaction, and the recombined pGWB5 plasmid was transferred into *Agrobacterium tumefaciens* strain EHA105 using the freeze/thaw method. To achieve its transient transformation in the *N. benthamiana* leaf, a 0.1 mL aliquot of the overnight *Ag. tumefaciens* culture was seeded into 10 mL yeast extract peptone medium containing 50 mg/L of both kanamycin and rifamycin. The cultures were shaken (200 rpm) and held at 28 °C until their OD_600_ reached about 0.5. The cells were then harvested by centrifugation (2000× *g*, 20 min), rinsed in 50 mM MES/KOH (pH 5.6), 2 mM Na_3_PO_4_, 28 mM glucose, 100 mM acetosyringone, then re-suspended in the same buffer to an OD_600_ of 1.0. The resuspended cells and p19 were mixed in a 1:1 ratio and the mixture incubated for 3 h at 25 °C, then infiltrated with a syringe into the base of a *N. benthamiana* leaf [39]. After two days, the fluorescence of the leaf hypodermis was inspected by laser confocal microscopy (Model LSM 700, Carl Zeiss AG, Oberkochen, Germany). The micrscope was equipped with both 495–570 nm (GFP) and 650–760 nm (chlorophyll) band pass filters. Image data were visualized using ZEN 2009 software (CarlZeiss AG).

## 4. Conclusions

This represents the first report of the in vitro functionality of a bryophyte HFT. The identification of a member of the BAHD family in as basal a land plant as the liverwort *M. emarginata* may reveal new insights into the diversity of acyltransferases.

## Figures and Tables

**Table 1:** Activity and Kinetics of Recombinant MeHFT on Different Acyl Donors and Acceptors.

Chemicals	Specific Activity (%)	K_m_ (μM)	V_max_ (nmol mg^−1^ min^−1^)	K_cat_ (s^−1^)	K_cat_/K_m_ (M^−1^s^−1^)
Acyl donor					
Feruloyl-CoA	100*	79.4 ± 10.86 ^a^ 83.23 ± 18.70 ^b^	83.23 ± 18.70 ^a^ 290 ± 37.49 ^b^	0.25263945 ± 0.015 ^a^ 0.250995 ± 0.032 ^b^	3181 ^a^ 3015 ^b^
p-Coumaroyl-CoA	38				
Caffeoyl-CoA	43				
Acyl acceptor					
1-Hexanol	31				
1-Heptanol	50				
1-Decanol	100 *	65.5 ± 11.21	297.3 ± 21.04	0.25731315 ± 0.018	3928
1-Dodecanol	89				
1-Tetradecanol	85				
1-Hexadecanol	n.d.				
16-Hydroxypalmitic acid	71				
Palmitic acid	n.d.				
1-Eicosanol	n.d.				
1-Docosanol	n.d.				

* Specific activity with feruloyl-CoA and 1-Decanol (102.34 ± 3.75 nmol mg^−1^ min^−1^) was taken to be 100%. n.d, not detectable. ^a^ Reactions were performed using feruloyl-CoA as the acyl donor and 1-Decanol as the acyl acceptor; ^b^ Reactions were performed using feruloyl-CoA as acyl donor and 1-Dodecanol as the acyl acceptor.

**Table 2:** The primers used in the present Investigation.

Primer Name	Primer Sequences (5’ to 3’)
MeHFT-F	ATGGGCGCCGAGGAAGTG
MeHFT-R	CAGCAGGCAGACGGAATT
MeHFT-pet32a-F	CGAGCTCATGGGCGCCGAGGAAGTGGT
MeHFT-pet32a-R	CCCTCGAGTTACAGCTTGATAGCTTCTT
attB1-MeHFT-F	GGGGACAAGTTTGTACAAAAAAGCAGGCTTAACCATGGGCGCCGAGGAAGTGGT
attB2-MeHFT-R	GGGGACCACTTTGTACAAGAAAGCTGGGTCCAGCTTGATAGCTTCTTCGA
